# A comparison of three‐film analysis software for stereotactic radiotherapy patient‐specific quality assurance

**DOI:** 10.1002/acm2.14203

**Published:** 2023-11-08

**Authors:** Athreya Buddhavarapu

**Affiliations:** ^1^ GenesisCare Epping Victoria Australia

**Keywords:** medical physics, quality assurance, radiochromic film, stereotactic radiotherapy

## Abstract

**Aim:**

The aim of this study was to investigate the suitability of three radiochromic film analysis software for stereotactic radiotherapy patient‐specific quality assurance (PSQA): FilmQA Pro v5.0, SNC Patient v6.2, and eFilmQA v5.0.

**Methods:**

Film calibration was conducted for each software followed by three sets of measurements. The first set assessed calibration accuracy by comparing measured and delivered doses at increments different from those used for calibration. The second set used each software to conduct PSQA through gamma analysis on 10 stereotactic radiosurgery (SRS) and stereotactic body radiation therapy (SBRT) patients. The third set utilized SNC Patient and eFilmQA to carry out gamma analysis on a collection of four digital test images, eliminating delivery and scanning uncertainties from impacting the analysis. Key supporting features within each software for conducting gamma analysis were identified.

**Results:**

Overall, FilmQA Pro and eFilmQA were deemed comparable and favoured over SNC Patient due to the presence of key features such as triple‐channel dosimetry, auto‐optimization, and dose scaling. FilmQA Pro has a substantial user base and established reputation. eFilmQA, having been introduced more recently, serves as a viable alternative to FilmQA Pro, having been further refined for stereotactic radiotherapy PSQA.

**Conclusion:**

This study investigated the suitability of three film analysis software (FilmQA Pro, eFilmQA, and SNC Patient) for stereotactic radiotherapy PSQA. Results from the investigation indicated that both FilmQA Pro and eFilmQA are comparably suitable and are preferred over SNC Patient. Both FilmQA Pro and eFilmQA are recommended for radiotherapy clinics.

## 1 INTRODUCTION

Stereotactic radiosurgery (SRS)/stereotactic body radiation therapy (SBRT) are advanced radiotherapy techniques using hypofractionated treatment schedules. As with intensity‐modulated radiotherapy (IMRT) and volumetric‐modulated arc therapy (VMAT), the Multi‐Leaf Collimator (MLC) is used to modulate beam intensity. PSQA is recommended for SRS/SBRT treatments to identify dosimetric discrepancies between planned and delivered treatments, arising from errors such as^1^:

- MLC leaf position errors (random and systematic).
- Gantry rotation errors.
- Couch motion errors.
- Beam stability errors (flatness, symmetry, output, dose rate).
- Undeliverable plans (e.g., high degree of modulations, small fields, and geometric localisation of dose distribution‐related problems).

A 2019 survey revealed that 15% of SRS/SBRT PSQA cases utilize Radiochromic Film (RCF), as opposed to other detectors.^2^ RCF measures the 2D dose distribution for a specific treatment field, which can be compared with the planned distribution in the Treatment Planning System (TPS). While alternative dosimeters, such as electronic portal imaging devices and diode arrays, can also measure 2D dose distributions, RCF is preferred for SBRT/SRS plan QA because it offers higher spatial resolution compared to other dosimeters, which are constrained by detector spacing.^3^

RCF consists of a radiosensitive dye that undergoes polymerization and absorbs optical light within the red, green, and blue spectrum upon exposure to ionizing radiation.^4^ This process causes the film to darken visibly. The extent of darkening can be calibrated against the absorbed dose, enabling radiation dosimetry.

Gafchromic™ EBT3 is a specific type of RCF featuring a unique configuration where polyester substrates are attached to both sides of the radiosensitive dye (active layer) to protect against mechanical damage and minimize effects from light exposure.^4^ Gafchromic™ EBT‐XD is a more recent evolution of EBT3 film, in which the active layer's crystal size has been reduced, making it less sensitive. Consequently, the dose‐response curve becomes steeper at doses above 10 Gy, rendering it more suitable for measurements above 10 Gy (e.g., SRS/SBRT).^4^

Calibration, readout, and analysis of RCF require a suitable flatbed scanner and film analysis software. Several commercially available software packages for scanning RCF exist, including third‐party software such as FilmQA Pro v5.0 (“FQP,” Ashland Global Specialty Chemicals Inc, Wilmington, Delaware, USA), SNC Patient v6.2 (“SNC,” Sun Nuclear Corporation, Melbourne, Florida, USA), and eFilmQA v5.0 (“EFQ,” IsoAnalytics Pty. Ltd. Melbourne, Victoria, Australia), which are used in this study. Each software possesses unique features to support the calibration and analysis of RCF. The user experience varies significantly among each software, making a comparison valuable. To date, there has been limited investigation into comparing RCF software.^5^ Such a comparison is important, as RCF is commonly used by radiotherapy clinics for stereotactic PSQA. The results of this study will enable recommendations to be made regarding which software would best address the needs of radiotherapy clinics.

## 2 METHODS

### 2.1 EBT3 and EBT‐XD RCF

This study utilized both GafChromic EBT3 and EBT‐XD RCF (Ashland).^6^ Originally purchased as 8“ x 10″ sheets”, the films were cut to fit the CIRS Multi‐Lesion Brain QA Phantom (Model 037, W: 150 mm x H: 190 mm x L: 170 mm)^7^ using a guillotine paper cutter. Each RCF offers submillimeter resolution (< 25 μm).^6^

### 2.2 Flatbed scanner

An EPSON 10000 XL flatbed scanner was used for the digitisation of RCFs with parameters set as follows^4^:

- 48‐bit color.
- Dpi‐resolution of 75 (0.35 mm pixel size).
- No colour correction.
- TIFF file format.

Before scanning, five mock scans were conducted to ensure scanner equilibrium.^8^ The orientation of the films during calibration and PSQA measurements was maintained to control for light polarization.^4^, ^9^ The film was consistently scanned in a “portrait” orientation, with the long side of the RCF sheet parallel to the scan direction.^4^ All scans were conducted with the film positioned at the centre of the scan region, at a consistent distance from the scanner borders to control for the lateral response artifact.^10^ Film was scanned 17 h post‐irradiation to match the time of calibration and control for post‐irradiation polymerization.^4^, ^11^

### 2.3 Film analysis software

Three film analysis software were investigated: SNC (including the advanced film registration package), FQP, and EFQ. Each software is capable of film dose calibration, film dose readout, comparison of the film 2D dose plane to TPS dose plane, and report generation.

FQP and EFQ can perform triple‐channel dosimetry to mitigate uncertainties from non‐dose‐dependent disturbances to the film.^12^ In contrast, SNC is only capable of single‐channel dosimetry. Both FQP and EFQ enable dose scaling, allowing for a recalibration of the calibration curve using a reference film with a known dose.^4^ FQP and EFQ also offer “auto‐optimization” (optimization), which involves computationally performing small translations and rotations of the film dose plane to improve alignment with the TPS dose, thereby improving the gamma pass rate.

### 2.4 Film calibration measurement

The film was cut into 19, 50 mm x 50 mm squares. Plastic Water (CIRS) was set up for irradiation using a beam energy of 6 MV FFF on a Varian TrueBeam linac, with the film placed at a depth of 1.2 cm (dose maximum) along the central axis within the plastic water slabs. This depth was chosen as the absorbed dose to water based on delivered MU is known. 10 cm of backscatter was placed under the film. The SSD to the top of the water slabs was 100 cm. A field size of 10 cm x 10 cm was used for all irradiations. Each piece of film was irradiated with a unique number of MU to cover the range from 0 to 30 Gy. The delivered dose was scaled against the daily output measured with a Farmer chamber in solid water.

### 2.5 Calibration curve generation

#### 2.5.1 FQP calibration

Calibration with FQP is conducted through the calibration process tree.^13^ Calibration films are imported as TIFF images, and a selection tool enables defining a region of interest (ROI) within the image. The ROI area measured 2 cm x 2 cm and was positioned within the centre of the scanned calibration film. Triple‐channel dosimetry was utilized, and the mean pixel density over the ROI area was recorded. A calibration table was generated using the mean pixel densities and delivered dose values. A reciprocal linear function was employed to fit the calibration table data.

#### 2.5.2 SNC calibration

In SNC, calibration is achieved by importing the calibration films as TIFF images and selecting a point in the image. SNC then automatically delineates an ROI around the selected point. The ROI size is not specified and cannot be altered. User‐provided dose values, in conjunction with mean pixel densities within the ROI, are used to generate the calibration curve. An exponential fit was used to fit the calibration data.

#### 2.5.3 EFQ calibration

EFQ features “SmartCal,” which streamlines the process of generating the calibration curve.^14^ SmartCal uses an image mosaic to automatically designate an ROI (∼2 × 2 cm) on all calibration film images and, in conjunction with user‐provided dose values, automatically generates the calibration curve. Triple‐channel dosimetry was used along with a rational fitting function to generate the calibration curve.

### 2.6 Calibration curve accuracy

The accuracy of calibration curves generated by each Film QA software was investigated by irradiating EBT‐XD film taken from the same batch used for calibration. Irradiations were within the same dose range used for calibration and with the same setup, but the delivered doses were different from those used for calibration. The size and position of the ROIs were kept consistent across all three software and matched those used for calibration. Single‐channel dosimetry was used with SNC, and triple‐channel dosimetry was used with FQP and EFQ. The red channel was used with all three software. Mean doses over the ROI, as recorded by each software for the test films, were compared against the known delivered doses.

### 2.7 SRS/SBRT treatment plan preparation

**FIGURE 1:**
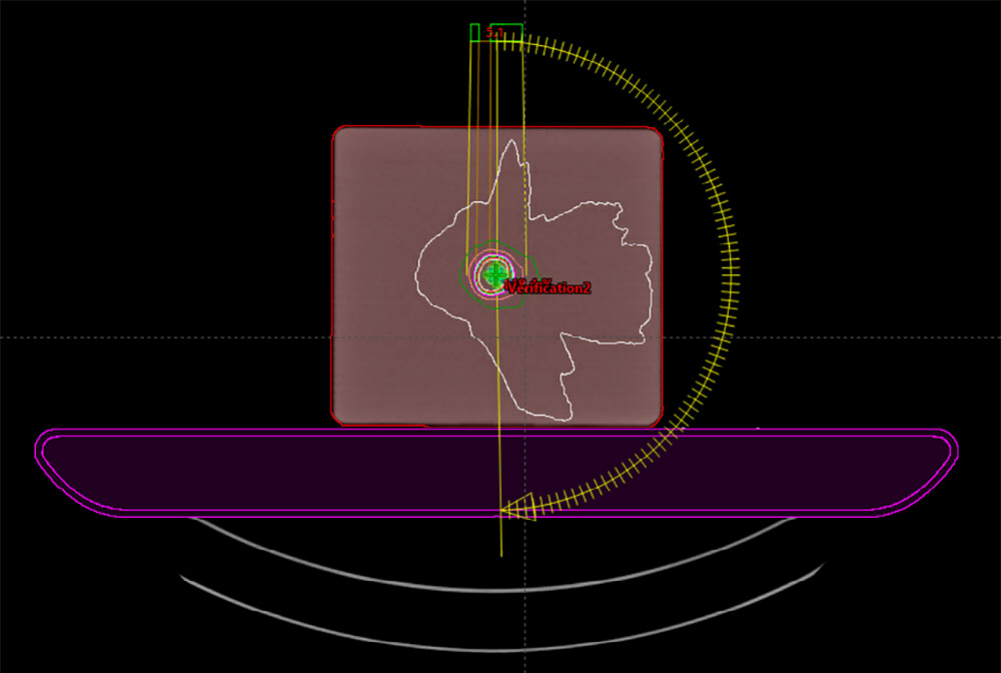
Simulation of the multi‐lesion phantom in the TPS for PSQA with film.

Treatment plans in this study were based on actual patient cases, involving SRS/SBRT treatments for various sites such as the lung, brain, spine, and pelvis. All plans were created using the Eclipse TPS v16.1 (Varian Medical Systems, Palo Alto, USA) and utilized the Acuros XB algorithm v16.1.0 and planned for delivery on the Varian TrueBeam linac using 6 MV FFF, VMAT, a 1400 MU/min dose rate, and a 0.125 cm dose calculation resolution. For measurement with film, plans were recalculated on the CIRS Multi‐Lesion Brain QA Phantom, centred at the treatment isocentre. 2D coronal dose planes passing through the PTV were exported for comparison against film measurements. Figure 1 illustrates the multi‐lesion phantom simulation in the TPS.

All plans were analysed using the three film analysis software. There were 10 plans in total, comparing SNC, FQP, and EFQ, using no dose scaling, optimization, and red channel analysis.

### 2.8 Treatment plan measurement and scanning

**FIGURE 2:**
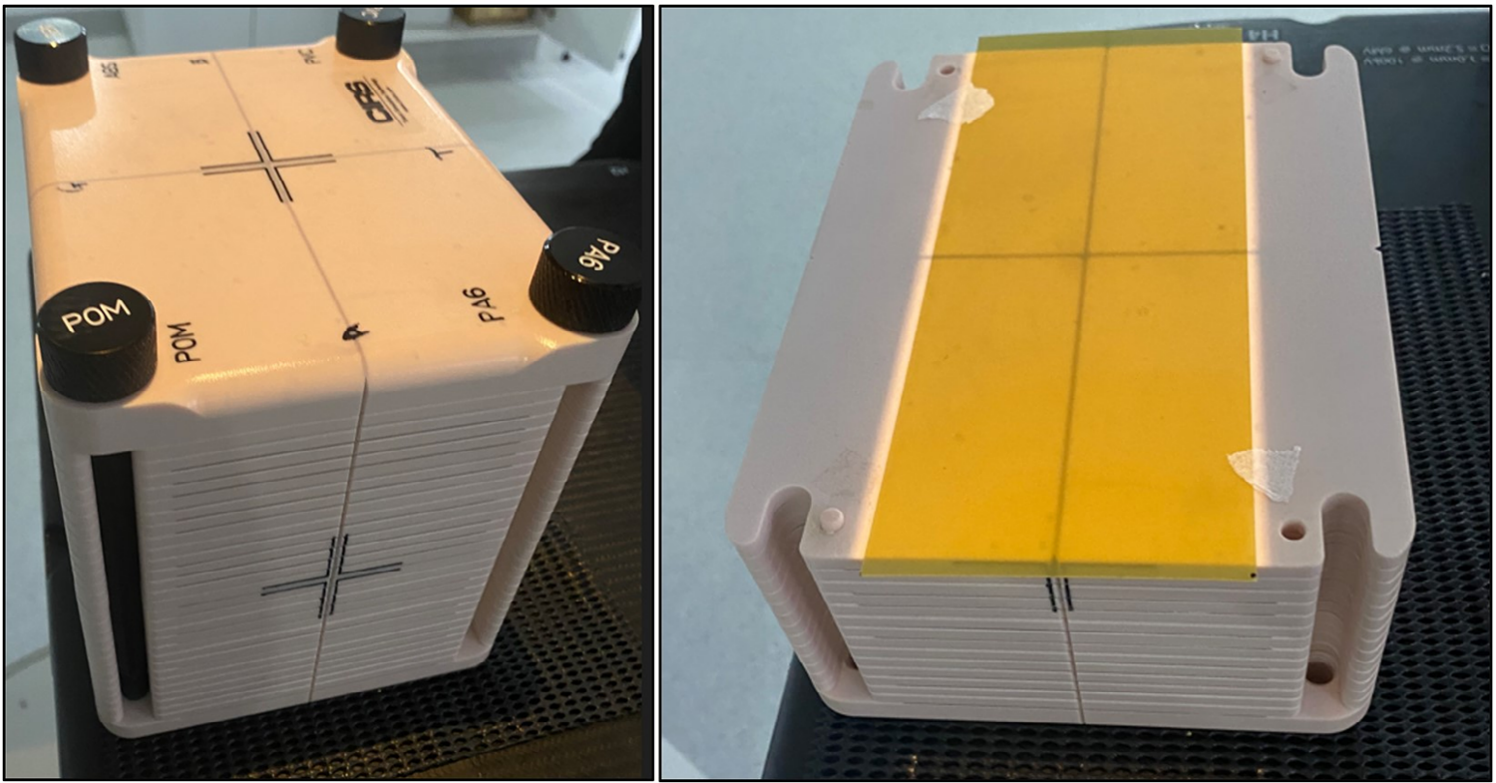
Setup of the multi‐lesion phantom with film for measurement.

Film was placed within the multi‐lesion phantom to match the depth of the exported dose planes. Figure 2 depicts the placement of film in the multi‐lesion phantom.

Markings on the film ensured that the orientation of the film on the multi‐lesion phantom could be kept consistent with that on the scanner. The multi‐lesion phantom, together with the film, was irradiated according to the treatment plan in a true composite measurement. Scanned images had ROIs delineated in preview mode to only include the film and were saved in TIFF format.

### 2.9 Treatment plan analysis film versus TPS

The gamma analysis method was employed to evaluate the agreement between the planned and delivered dose distributions.^15^ The gamma method calculates a unitless “gamma index” value for points within the measured (or referenced) dose distribution. The planned dose distribution served as the reference, with the film distribution being assessed against it.

The gamma criteria for all plans was:

- 5%/1.5 mm for lung/pelvis/spine.
- 5%/1 mm for brain/brain stem.

The dose threshold was set at 10%. The pass rate for all plans was 95%.

### 2.10 Comparison to TPS with SNC/FQP/EFQ

#### 2.10.1 FQP

The “dose map” and “dose to plan comparison” modules in FQP enable loading and comparing the film dose to the TPS dose.^13^ After loading the film dose as a TIFF image, registration against the TPS dose can be performed by identifying fiducial markers on the film, from which an isocentre location is derived. An ROI on the film is delineated to only include the central high‐dose region (50% isodose). An ROI for the TPS dose distribution was also delineated to only include the central high‐dose region. Finally, gamma analysis is used to compare the measured and planned dose distributions.

#### 2.10.2 SNC

Film images are loaded as TIFF images. Fiducials can then be identified for registration against the TPS dose distribution. After registration, the film dose is saved as a “.flm” file. Comparison against the TPS dose distribution proceeds similarly to how it is done with other SNC equipment such as the MapCHECK and ArcCHECK.

#### 2.10.3 EFQ

The steps required to produce results are as follows: Load the calibration curve, load the film dose distribution (TIFF image), load the TPS dose distribution, register the film and TPS dose distributions by identifying the isocentre through fiducial markers, crop the film dose to remove markings and include the ROI, perform gamma analysis, and generate the report.^14^

### 2.11 Comparison with digital test images

A suite of test images, freely available for online download, was used to evaluate the gamma analysis within EFQ and SNC.^16^ These test images were previously used to compare gamma results from different software and serve to provide a frame of reference for comparing results. The digital nature of the images removes compounding factors such as delivery fluctuations, detector geometry, detector resolution, and setup errors from influencing the gamma results.^16^ The suite of test images comprises four image sets, each including an “evaluated” and “reference” image. A summary of the image sets is provided in Table 1.

**TABLE 1:** Summary of digital image sets.

Test image set	Description
Geometric Test (DTA)	The evaluated images consist of a square identical in size and dose, offset laterally (50 px) and vertically (200 px) by a fixed distance.
Geometric Test (DD)	The evaluated images consist of a square in the same position and size with a dose offset of 1.2%.
IMRT Field	A simple prostate IMRT plan was created using the TG‐119 test suite. The image has a modulation complexity score of 0.52.
VMAT Field	A complex head and neck VMAT arc. The image has a modulation complexity score of 0.14.

More information on the test images can be found in the original paper by Agnew et al.^16^ Images were analysed using each software with the same gamma criteria used in that paper:

- Global dose normalisation to the maximum dose in image “reference.”
- Local dose normalization was used when global was not available.
- A 1%/1 mm gamma criteria.
- The images came with a pixel size of 0.25 mm.
- 10% minimum dose threshold.
- No image alignment was performed.
- Noise removal used with EFQ.

All image sets were analysed with SNC and EFQ. Image sets could not be analysed with FQP, as they did not come packaged with associated calibration curve data, which is necessary for analysis with that software.

## 3 RESULTS

### 3.1 Film calibration accuracy

**FIGURE 3:**
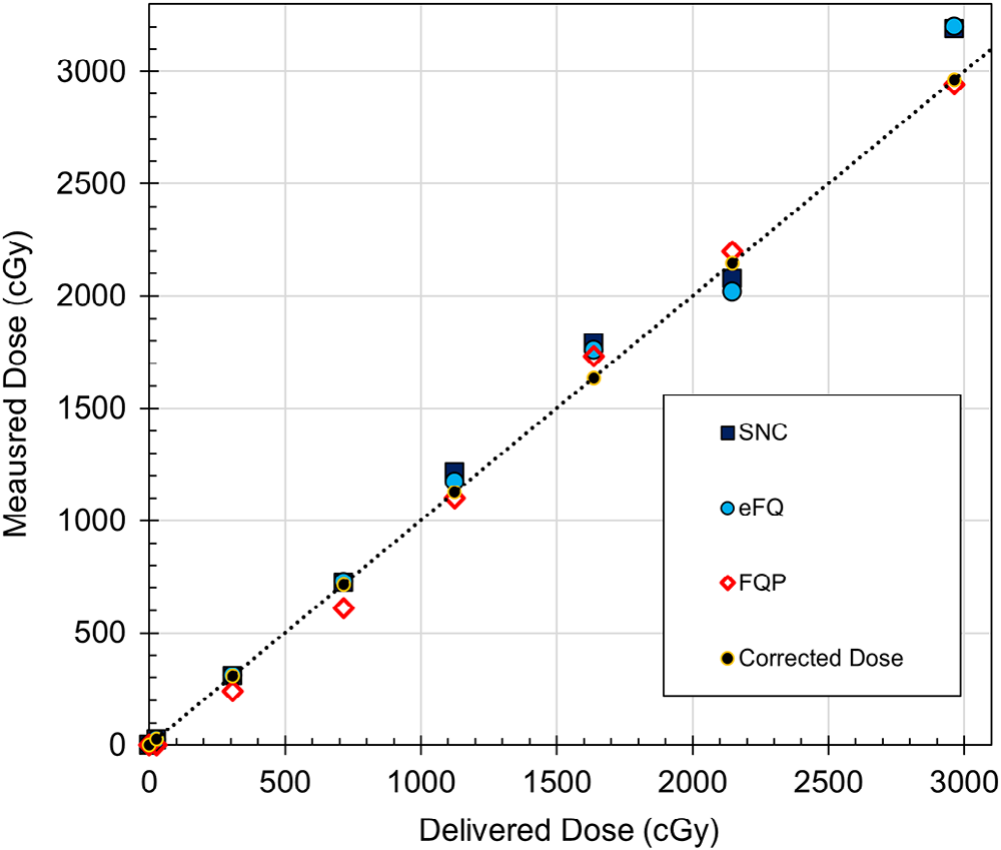
Results of calibration accuracy test comparing measured and delivered (corrected) doses across all three software.

Figure 3 depicts the results of the calibration accuracy test. The corrected dose represents the delivered dose (scaled by the daily output), and the film dose is taken to be that reported by the film software. EFQ and SNC demonstrated comparable percentage deviations, with maximum percentage deviations of −10.0% and 9.5%, respectively. However, for EFQ, this maximum was observed at a relatively low dose of 25 cGy, resulting in a difference in absorbed dose of 2 cGy. In this context, the second‐highest percentage deviation for EFQ (8.0% at 2964 cGy) is more clinically significant, because it translates to an absorbed dose difference of 236 cGy.

In contrast to EFQ and SNC, FQP exhibited higher percentage deviations, with a maximum of −21.4% for the corrected dose of 307 cGy. This excludes the percentage deviation for the lowest delivered dose (25 cGy) which was considered an outlier. Although percentage deviations with FQP were relatively smaller at higher doses (≥1124 cGy), they were larger at lower doses (<1124 cGy).

### 3.2 SRS/SBRT treatment plans

**FIGURE 4:**
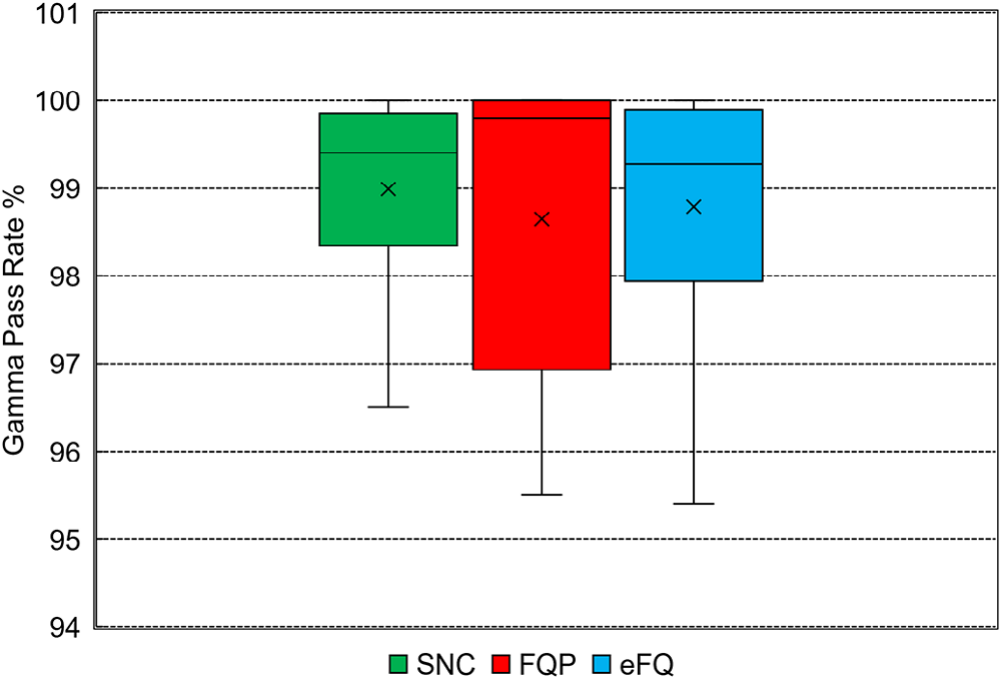
Box and whisker plot of gamma pass rates for SRS/SBRT plans.

A statistical evaluation of the gamma pass rates with each software is shown in Figure 4.

Table 2 summarizes key statistics from the analysis of the patient plans. The difference in median and mean pass rates among the three software was within 0.5%, indicating good agreement. The largest deviation among the three software was found in the lower interquartile range, with FQP reporting a notably lower IQR of 96.9% compared to more similar values for SNC and EFQ (98.3% and 97.9%).

**TABLE 2:** Key statistics from analysis of patient plans.

Film software	Median	Interquartile range	Mean
SNC	99.4%	98.3–99.8%	99.0%
FQP	99.8%	96.9–100%	98.6%
EFQ	99.3%	97.9–99.9%	98.8%

### 3.3 Digital test images

**FIGURE 5:**
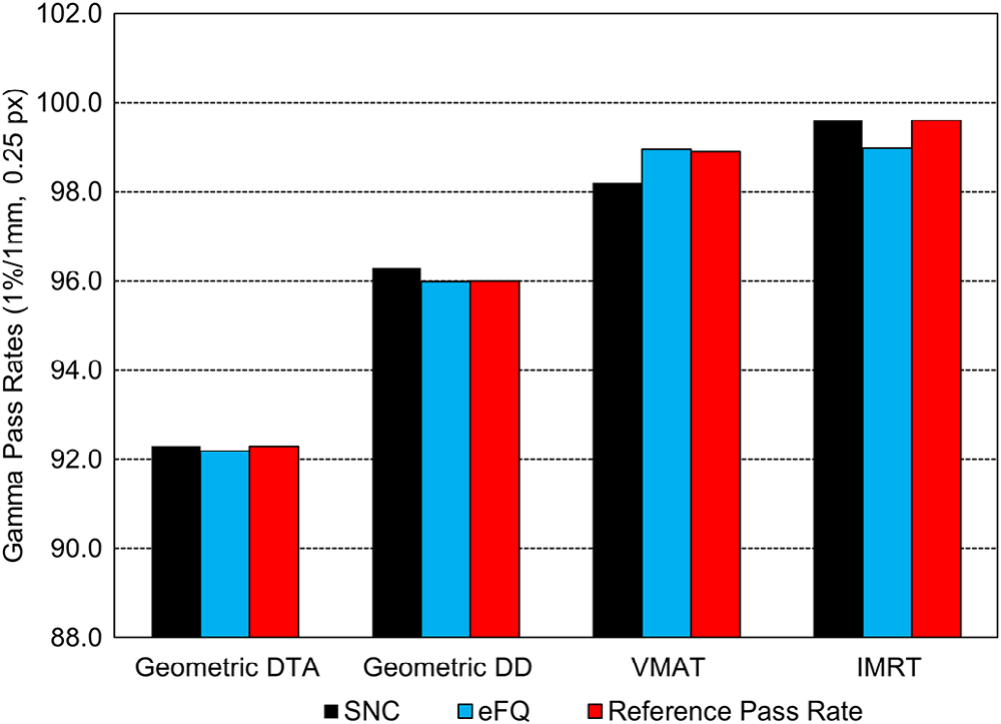
Gamma pass rates for digital images.

Figure 5 illustrates the pass rates for SNC and EFQ for each of the test image sets. The reference pass rate is the manually calculated pass rate for the DD and DTA tests and the median pass rate of 10 different gamma analysis software used in the original study by Agnew et al. for the IMRT and VMAT tests.^16^

Percentage differences between the reference pass rate for the DTA test were 0.1% and 0% for EFQ and SNC, respectively. Similarly, the percentage difference to the reference pass rate for the DD test was 0.02% and −0.3% for EFQ and SNC, respectively.

For the VMAT field, the percentage difference to the reference pass rate was −0.07% and 0.7% with EFQ and SNC, respectively. Likewise, for the IMRT field, the percentage difference to the reference pass rate was 0.61% and 0% with EFQ and SNC, respectively.

## 4 DISCUSSION

### 4.1 Film calibration curve accuracy

Both SNC and EFQ reported comparable accuracy, while FQP demonstrated relatively improved accuracy at higher dose levels and unexpectedly poor accuracy at lower dose levels. These results differ from those in literature. Howard et al. investigated the accuracy of film calibration for FQP and Image J,^17^ reporting a maximum percentage difference of 5%, with the majority of results within 2% over a delivered dose range of 0–3000 cGy when measured with both EBT3 and EBT‐XD film. As previously mentioned, all three film analysis software use different fitting functions, and calibration uncertainties are influenced by this. A recent article by Rodriguez^18^ indicates that uncertainties related to the fitting process during calibration can vary by a factor of two, depending on the fitting function applied. To reduce fitting uncertainties, the calibration process could be repeated using a larger number of calibration points.^18^, ^19^ The “PDD method” is an alternative method for performing film calibration and allows for the rapid collection of multiple calibration points.^19^ As follow‐up work, the PDD method is suggested to better assess film calibration accuracy.

Another consideration is the use of triple‐channel dosimetry with FQP and EFQ, compared to single‐channel dosimetry with SNC. A study by Hayashi^20^ found that uncertainties in calibration are reduced in the lower dose range when using the triple‐channel method as opposed to the single‐channel method. Specifically, the maximum standard deviation was reduced by half using triple‐channel dosimetry.^20^ This suggests that follow‐up work should also investigate the impact of triple‐channel versus single‐channel dosimetry on calibration uncertainty.

### 4.2 SRS/SBRT treatment plans

Plans tended to pass at similar rates among all three software with the mean and median passing rates agreeing within 0.5%. FQP showed the highest dispersion in pass rates followed by EFQ. A limitation of these results is that only 10 plans were analysed using just the red channel.

The implementation of the gamma algorithm cannot be excluded as a factor influencing pass rates.^1^ Therefore, results from these plans are not reliable indicators of which software provides more accurate comparisons to the TPS.^16^ However, they can suggest which software is best suited for radiotherapy quality assurance when considering factors such as the user experience and available features, which is discussed in subsequent sections.

### 4.3 Digital test images

Results using these digital test images are more relevant to how accurately each software is comparing the planned and measured dose distributions.^16^

#### 4.3.1 Geometric DTA and DD tests (1%/1 mm, 0.25 px)

For accurate results using a gamma criteria of 1%/1 mm, the pixel pitch should be < (DTA Distance/3).^15^ As such, the used pixel pitch of 0.25 mm meets this requirement and can be relied upon when using this gamma criteria. Results for SNC had already been investigated in the original paper by Agnew et al.; however, results for EFQ had not been investigated before this work.^16^ Both SNC and EFQ remained within 0.5% of the manually calculated pass rates, with SNC reporting the largest percent deviation of 0.3% for the DD test.

#### 4.3.2 IMRT/VMAT tests (1%/1 mm, 0.25 px)

Both SNC and EFQ remained within 1% of the median pass rate of 10 alternative film analysis software, presented by Agnew et al.^16^ Additionally, EFQ remained within the IQR for all software, while SNC was just under the lower IQR for the VMAT field.

### 4.4 Comparison of features between SNC FQP and EFQ

Some features important to film analysis are not supported by all three software. The importance of dose scaling, optimization, and triple‐channel dosimetry makes SNC the least favourable for PSQA since it lacks these features. This leaves FQP and EFQ.

Triple‐channel dosimetry aims to separate dose‐dependent contributions to the film response from any non‐dose‐dependent disturbances.^4^, ^12^ Non‐dose‐dependent disturbances include variations in film thickness, artifacts, and scanner readout noise. Removing these disturbances has been found to improve film dosimetry and analysis.^20^, ^21^

Scaling the treatment film dose distribution helps mitigate uncertainties between calibration and measurement. Commonly referred to as the “one scan protocol,” a reference film is irradiated by a known dose (typically the plan maximum) and used for recalibrating the treatment film dose.^4^ This process can help reduce uncertainties due to interscan variability, environmental conditions, and post‐irradiation optical density growth (reducing the wait time between irradiation and readout).^4^ ^.^ ^22^

Registration between the film and TPS dose is performed by manually identifying fiducial markers on the film with all three software. However, even after manual registration, small misalignments between the film and TPS dose distributions commonly remain. In these circumstances, these misalignments can be corrected with the ‘optimization’ feature available in FQP and EFQ.^13^, ^14^ Optimization involves performing small translations and rotations to the film dose to improve alignment with the TPS dose, improving the gamma pass rate. Generally, optimization takes longer with FQP than EFQ. These translations and rotations can also be performed in SNC but must be done manually, which takes considerably more time. Table 3 summarizes important film analysis features within each software.

**TABLE 3:** Key film analysis features within each software.

Analysis features	SNC	FQP	EFQ
Dose scaling	⨯	✔	✔
Optimization	⨯	✔	✔
Triple‐channel dosimetry	⨯	✔	✔
Temporal calibration (time‐independent dosimetry)^23^	⨯	⨯	✔
Save case	✔	✔	⨯
Micro manual adjustments (translational/rotational)	✔	✔	⨯
Workflows (automated multi‐film analysis)	⨯	⨯	✔

All three software can generate reports. FQP can produce the largest variety of charts and graphs, followed by EFQ, and then SNC. Each software offers a free trial/demo version that clinics can try before committing.

### 4.5 Suitability of each software for radiotherapy PSQA

The overall suitability of each film analysis software for radiotherapy PSQA was assessed holistically. To determine the suitability for PSQA, the following factors were considered for each software:

- Processing time for results.
- Learning curve.
- Reporting features.
- Analysis features.

The learning curve, processing time, reporting features, and analysis features were all important factors when assessing the suitability of each software for PSQA. The learning curve is related to the number of steps required to generate results. In this context, FQP has the steepest learning curve due to its numerous features and steps for conducting film analysis and creating a report. With FQP's extensive user interface, users must consult the manual and learn which buttons to use, and in which order to analyse results. Many features in FQP are not essential or relevant for PSQA. Both SNC and EFQ have similar learning curves, as the process of registering the film and TPS dose, processing the gamma analysis, and creating a report involve a comparable number of steps. While new users need to refer to the manual for PSQA with EFQ and SNC, EFQ's “Workflows” feature significantly simplifies the required steps by guiding users through the process step by step and automating the process of analysing multiple films.

The number of steps needed for PSQA is also strongly related to the time it takes to process results and create a report. EFQ is the quickest among the three software at generating results, mainly due to its optimization feature, SmartCal and Workflows, which make it noticeably quicker than FQP. Specifically, optimization typically takes seconds with EFQ compared to minutes with FQP.

A key disadvantage of EFQ compared to the other software is the user interface, which can be described as relatively rudimentary. For example, one feature known as “manual keep” is used to remove fiducial markings on the RCF to prevent them from interfering with the analysis, operating similar to a crop. After users define the ROI to be cropped using this feature, they must double‐click on the ROI to perform the crop. However, the requirement to double‐click is not communicated within the user interface, which necessitates consulting the manual to be aware of it. Another example is the need to reselect the chosen RCF and TPS dose files from the dropdown list to proceed with the analysis, a step only communicated in the manual. The capability to save a specific case and allow users to reload their work is also absent in EFQ. These minor inconveniences steepen the learning curve and contribute to why EFQ and FQP are considered comparable at this juncture. Addressing these issues in future updates could make EFQ a promising choice as the preferred film analysis software for stereotactic radiotherapy PSQA.

## 5 CONCLUSION

This study examined three film analysis software (FQP, EFQ, and SNC) and compared their suitability for stereotactic radiotherapy PSQA. Each software was evaluated for calibration curve accuracy, treatment plan analysis, and reporting capabilities. Both FQP and EFQ possess key features that support film analysis, such as triple‐channel dosimetry, auto‐optimization, and dose scaling, making them preferable over SNC. FQP and EFQ were found to be comparably suitable. FQP is a widely adopted and reliable software for film analysis, with a large user base and an extensive history. EFQ, on the other hand, serves as a comparable alternative to FQP, retaining essential features for accurate film analysis while also being further refined for stereotactic radiotherapy PSQA. Both FQP and EFQ are recommended for radiotherapy clinics. Clinics may try a free trial or demo version of either option before making a commitment.

## AUTHOR CONTRIBUTIONS

The author confirms sole responsibility for the following: Study conception and design, data collection, analysis and interpretation of results, and manuscript preparation.

## CONFLICT OF INTEREST STATEMENT

The author declares no conflicts of interest.

## ETHICS STATEMENT

This article does not contain any studies with human participants or animals performed by any of the authors.
